# The inequalities and challenges of prehabilitation before cancer surgery: a narrative review

**DOI:** 10.1111/anae.16502

**Published:** 2025-01-08

**Authors:** Hilary Stewart, Sophie Stanley, Xiubin Zhang, Lisa Ashmore, Christopher Gaffney, Jo Rycroft‐Malone, Andrew F. Smith, Laura Wareing, Cliff Shelton

**Affiliations:** ^1^ Lancaster Medical School Lancaster University Lancaster UK; ^2^ North West School of Anaesthesia Manchester UK; ^3^ Faculty of Health and Medicine Lancaster University Lancaster UK; ^4^ Department of Anaesthesia Royal Lancaster Infirmary Lancaster UK; ^5^ Department of Anaesthesia Wythenshawe Hospital Manchester UK

**Keywords:** cancer, oncology, prehabilitation, surgery

## Abstract

**Introduction:**

Prehabilitation seeks to enhance functional capacity and preparedness before surgery with the aim of improving outcomes; it is generally based on exercise, diet and psychological interventions. While there is obvious appeal to this approach in terms of patient experience and resource use, the interventions are complex and the evidence base for prehabilitation before cancer surgery is heterogeneous. Prehabilitation requires patient understanding and motivation as well as commitment of resources. Programmes are challenging to design and implement, and can generate ‘intervention‐based inequalities’ based on the capacity of patients to engage. We present a narrative review on the inequalities and challenges of prehabilitation before cancer surgery.

**Methods:**

We searched databases of peer‐reviewed research to identify appropriate articles. We used the results in combination with iterative searches based on citation tracking, grey literature (e.g. patient information resources) and articles from personal libraries, to develop our discussion.

**Results:**

We describe the uncertainties in the evidence base for prehabilitation before cancer surgery, and the challenges and barriers for healthcare providers, systems and patients. Key findings include that prehabilitation is under‐researched in many cancers and that people with lower health literacy, from minority ethnic groups and socio‐economically disadvantaged backgrounds, are less likely to engage, despite often having worse peri‐operative outcomes.

**Discussion:**

Prehabilitation must be implemented carefully to avoid widening inequalities. More research is needed, both in terms of the impact of interventions and to understand how prehabilitation should account for the social determinants of health.

## Introduction

Advancements in surgical practice alongside improvements to peri‐operative care and enhanced recovery pathways have contributed to improved outcomes for patients undergoing cancer surgery [1, 2]. Despite this, major surgery still puts many at significant risk of harm to their physical and mental health. Outcomes are predictably worse for patients who may be less able to withstand surgical stress, leading to prolonged hospitalisation, increased medical interventions, readmissions and poorer cancer outcomes, while also being costly to healthcare systems [1, 3, 4]. Rates of morbidity remain high, with postoperative complications affecting 15–40% of patients [2, 4, 5]. Adverse effects have traditionally been managed by clinical care teams and rehabilitation/intermediate care services, the demand for which will continue to increase as more people are diagnosed with, and survive, cancer [6]. This unsustainable position has led to an increasing interest in preventative measures to avoid complications and improve recovery.

Prehabilitation, although varyingly described across literatures, is broadly the practice of enhancing a patient's functional capacity before major interventions such as surgery, with the aim of improving postoperative outcomes. Representing a ‘paradigm shift’, whereby interventions that target postoperative outcomes are implemented before surgery, the pre‐operative period is reconceptualised as an opportunity to prepare [7, 8, 9, 10]. In the context of resource‐limited healthcare systems, the potential of prehabilitation to improve financial value, care quality and clinical outcomes has generated considerable enthusiasm [10, 11]. Accordingly, research has sought to identify interventions that enhance patients' capacity and reduce surgical risk, which has been characterised as a shift from ‘passive risk assessment’ in pre‐operative clinics to one of ‘active risk mitigation’ [7].

Theoretically, patients with cancer stand to benefit from prehabilitation that prepares them for the demands of surgery and other invasive treatments. However, the evidence base for prehabilitation in cancer care is inconsistent, making it challenging to put into practice. Moreover, little is known about what patients want from prehabilitation, or how their views are considered in the design and delivery of services, complicating how the value and benefits of prehabilitation are communicated. Given the potential for prehabilitation programmes to create inequities through ‘intervention‐generated inequalities’ (i.e. those which are based on a person's ability to engage with an intervention) [12], these should form part of research and be considered in implementation.

## Methods

The paper presents a narrative review on the inequalities and challenges of prehabilitation before cancer surgery. We searched electronic databases of peer‐reviewed research including PubMed, MEDLINE and Cochrane databases to identify appropriate articles. Keywords comprised ‘prehabilitation’; ‘inequality’; ‘cancer surgery’; and associated synonyms (prehab; pre‐operative rehabilitation; preconditioning; cancer; or malign* or carcinoma or neoplas*). We used the results, in combination with further iterative searches based on forward and backward citation tracking, the grey literature (e.g. patient information resources) and articles from personal libraries, to develop our discussion. Key themes across the reviews are summarised to describe current research and controversies.

## Results

**Figure 1:**
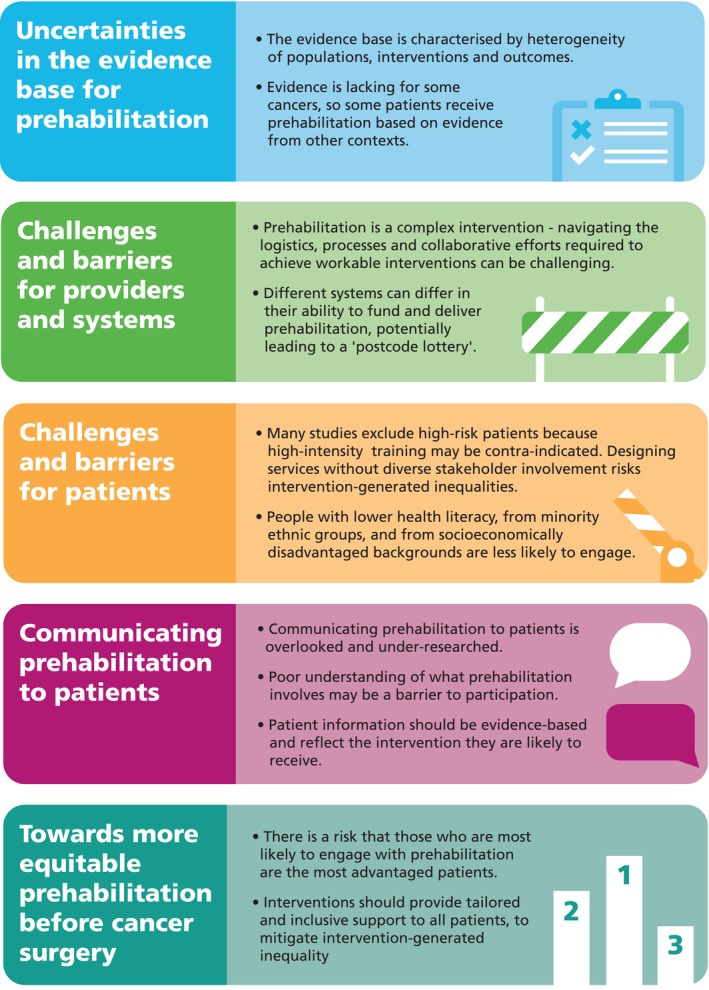
Infographic summarising findings and conclusions.

We begin with a review of the complex landscape of evidence for prehabilitation before proceeding to a review of the challenges and inequalities inherent to prehabilitation before cancer surgery and highlighting some underexplored areas where more research is needed (Fig. 1).

### Uncertainties in the evidence base for prehabilitation

The evidence base for prehabilitation in cancer care is characterised by heterogeneity across the populations, interventions and outcome measures used in research. Interventions have been delivered in various combinations (unimodal or multimodal); locations (hospital or home); and formats (face‐to‐face or digital) [13, 14]. Broadly, these interventions include exercise; nutritional; psychological; and behavioural components [14]. Evidence for some cancer types (e.g. colorectal, lung and upper gastrointestinal) is more comprehensive than for others (e.g. breast, pancreatic, haematological, head‐and‐neck or gynaecological) [13], meaning that some patients receive prehabilitation based on evidence extrapolated from other contexts.

Prehabilitation most commonly prepares patients for surgery, although more recently has been trialled before chemotherapy, radiotherapy and stem cell transplants [14, 15, 16, 17]. Multiple outcome measures have been used to assess the efficacy of prehabilitation. A scoping review reported that 184 different measures evaluated 50 different outcomes across 76 randomised controlled trials of prehabilitation interventions before surgery [18]. Attempting to synthesise the evidence across this complex landscape is, therefore, challenging. Few clear conclusions have been generated, with variable certainty [13, 14, 19].

There are some contexts in which the efficacy of prehabilitation has been shown. Multiple types of exercise‐based prehabilitation (aerobic, resistance and inspiratory muscle training) appear to reduce postoperative pulmonary complications and duration of hospital stay in patients with lung cancer undergoing thoracic surgery [20, 21, 22]; nutritional prehabilitation may improve rates of infectious complications and reduce duration of hospital stay in patients with colorectal cancers treated surgically [23, 24]; and short‐term improvements in dysphagia have been observed when swallowing exercises are delivered before treatment for head‐and‐neck cancers, although evidence of the impact of this on quality‐of‐life is conflicting [16, 25].

Outside of these contexts, the benefits of prehabilitation are less certain. For example, pelvic floor exercises before radical prostatectomy have not reduced rates of postoperative urinary incontinence consistently or been found to improve patients' quality‐of‐life [19, 26, 27, 28, 29]. It is also unclear whether exercise‐based prehabilitation improves pulmonary and postoperative outcomes in patients with upper gastrointestinal cancers [13, 30, 31, 32, 33]. Some reviews found pre‐operative exercise reduced postoperative pneumonia rates when interventional and observational study data were combined, suggesting that further high‐quality randomised trials may be required [31, 32, 33].

In patients with colorectal cancer, some reviews find that exercise prehabilitation improves fitness [13, 34], whereas others do not [35, 36]. Evidence suggests more consistently that exercise prehabilitation does not improve postoperative outcomes (duration of hospital stay, complication rate or mortality) for these patients [13, 23, 34, 35, 36]. The effects of nutritional and exercise interventions on postoperative outcomes in patients with colorectal cancer appear to differ. As such, when combined in multimodal programmes, it becomes challenging to interpret the overall effect [23, 24, 37, 38].

A recent systematic review and meta‐analysis of high‐quality randomised controlled trials on the role of prehabilitation programmes in colorectal surgery reported that prehabilitation was associated with better functional test results at the time of surgery and a shorter duration of hospital stay [39]. However, when compared with the usual standard of care, overall complications and readmission rates were similar. The authors note this as important when considering the value proposition of such an intervention, especially in lower‐resource healthcare systems which may struggle with the high costs of a multimodal prehabilitation programme [39]. When compared with exercise‐based rehabilitation, prehabilitation produced similar outcomes, which is consistent with a previous Cochrane review [35]. This result is important given the timeframes required to deliver prehabilitation and consequent potential for delays to surgery, when similar benefits can be achieved using rehabilitation after treatment [39].

Reasons for these inconsistent findings may include that individual cancers and their treatments are distinct and as such may respond differently to prehabilitation regimes, and that different cancers tend to occur in different demographic groups. Attempts to compare different exercise interventions (aerobic, resistance, respiratory or combined training) have not revealed a single optimum regime [13, 14, 19, 22]. However, comparisons are often made in heterogenous groups of patients (e.g. people with lung, gastrointestinal, breast and urological cancers) rather than focusing on interventions in a specific cancer type [19], and furthermore, it is known that responses to exercise and dietary interventions differ between individuals [40].

Prehabilitation literature has tended to assess efficacy using observer‐reported (duration of hospital stay, re‐admission or mortality) or clinician‐reported (complication rates) outcomes [18]. Fewer studies measure the impact of prehabilitation on patient‐reported outcomes although when this has been attempted, findings seem similarly inconsistent [26, 41] Some reviews find that exercise prehabilitation (not targeted at a specific complication) may improve quality of life [26, 41]; however, this is not consistent among other unimodal or multimodal intervention studies [19, 26, 42, 43]. Exercise prehabilitation also does not appear to improve postoperative symptoms of fatigue or insomnia, although few systematic reviews have commented on this [19, 21, 44]. The limited literature on patient‐centred outcomes in prehabilitation in turn limits clinicians' ability to conduct accurate consent conversations and help patients to weigh the pros and cons of participation.

In addition to uncertainties within the existing evidence, many aspects of the efficacy of prehabilitation remain under‐researched. Few systematic reviews involve patients with breast, pancreatic or haematological malignancies, and none are specific to patients with gynaecological cancer [13, 17, 45, 46]. Fewer than 15% of cancer surgery prehabilitation trials involve a psychological component, possibly because these interventions do not appear to improve traditional ‘surgical’ outcomes [23, 47, 48, 49]. This ignores the potential benefit that psychological interventions may have on patient‐reported outcomes, such as quality of life, pain symptoms or mood disturbances, and the significance of psychological factors for treatment compliance [26, 48, 49, 50]. For example, experiences of trauma may limit patients' capacity to engage with both prehabilitation and cancer treatment, and may be implicated in behavioural risk factors such as substance use [51].

### Challenges and barriers for healthcare providers and systems

Much of the evidence for prehabilitation is inconsistent and comes from heterogenous populations, and it is acknowledged that what is achieved in clinical trials does not necessarily translate into implementation in services [52]. As such, prehabilitation programme design tends to be pragmatic about what can be achieved in local settings. As a complex intervention comprising multiple components which must work interdependently in contexts where patients are often receiving other types of complex care (e.g. chemotherapy and radiotherapy), navigating the logistics, processes and collaborative efforts required to achieve workable interventions can be challenging for care teams and healthcare systems [3, 52]. Likewise, this presents a challenge for healthcare researchers and may be one reason why the evidence base shows such heterogeneity.

A study of prehabilitation services in Scotland revealed that many healthcare professionals were unaware of the availability of local services; when these services were described, there was a lack of clear definitions for prehabilitation and outcome measures, as well as varying referral processes [53]. A similar report from a qualitative study examining the perspectives of professionals involved in prehabilitation identified several significant barriers to implementation. These were primarily related to the intervention being complex, a lack of awareness of local prehabilitation provision and uncertainty regarding the potential benefits of prehabilitation [52].

Previous studies have also identified organisational barriers to implementation, such as long‐term funding, scalability and issues related to individual access to, acceptance of, and adherence to prehabilitation [54]. Other qualitative studies with healthcare providers have identified knowledge; resources; poor patient engagement; and inconsistent practice as barriers for professionals [54]. Other barriers include limited workforce capacity [53, 54, 55]; insufficient referral to prehabilitation [53, 56]; challenges in co‐ordinating cross‐boundary systematic service delivery [57]; and inadequate funding and resources [53, 56]. This means that different systems and localities may differ profoundly in their ability to fund and deliver prehabilitation, potentially leading to a ‘postcode lottery’ of access to services.

### Challenges and barriers for patients

Surgical patients of low socio‐economic status have increased duration of hospital stay, higher rates of postoperative complications and morbidity and reduced overall survival [58]. They also have higher rates of comorbidity at cancer diagnosis, which influences the timing, tolerance and outcome of treatment. Those who are most disadvantaged are less likely to be offered curative treatment, despite evidence that many would stand to benefit from such treatment [59].

Poor postoperative outcomes are associated with conditions and phenotypes which are known to be linked to low socio‐economic status, such as malnutrition, sarcopenia, low physical activity, anxiety and depression, and are worse for older adults and people with frailty [60, 61]. Despite having much to gain from new healthcare interventions, older patients and those with multimorbidity are routinely under‐represented in cancer clinical trials, limiting the applicability of trial results to these important groups of patients [59, 62, 63]. Indeed, many prehabilitation trials have excluded high‐risk patients on the basis that high‐intensity training may be too challenging or even contraindicated, and there are suggestions that those who enrol in clinical trials are the most motivated or able patients [40, 64]. This may also potentially contribute to a dilution effect in studies, possibly accounting for some inconclusive results and, as such, clearer definitions of the target population for prehabilitation would be beneficial [39]. Furthermore, participation in prehabilitation interventions is lower for people from socio‐economically deprived communities and some minority ethnic groups [61, 63]. The reasons for this are not well investigated at present but considering that many prehabilitation interventions require financial commitment (e.g. to purchase exercise clothing or healthier foods), travel (e.g. to attend hospital appointments or physical activity sessions at locations which are increasingly centralised) and the ability to communicate fluently in the languages used by the healthcare system, it is easy to see why this may occur. Studies also find that treatment and survival outcomes are poorer among these groups compared with socio‐economically advantaged and majority groups [58, 63].

Acceptance and adherence are critical factors in the effectiveness of prehabilitation [64, 65]. An umbrella review of surgical prehabilitation systematic reviews found only 36% of studies reported adherence rates, with a mean (SD) rate of 70% (24%) [14]. Qualitative research suggests that prehabilitation is broadly acceptable to patients, and indeed valued by many who can engage with it, but several barriers to uptake and adherence are noted [65, 66]. Factors affecting adherence to prehabilitation programmes negatively include patients' desire for expedited surgery; their self‐assessments of fitness; personal and professional obligations; health issues and physical symptoms; holidays, and the alteration of surgery dates [67, 68]. Barriers such as physical symptoms (e.g. nausea, pain and fatigue, which are common among people with cancer) and loss of motivation (e.g. due to poor emotional wellbeing or negative feelings towards exercise or diet) may hinder physical activity and healthy eating [68]; however, all these issues can potentially be mitigated in a sufficiently supportive and adequately resourced healthcare system. Other factors such as time constraints [68], inadequate resources to support engagement [61] and transportation issues [69] also serve as barriers to participation, while facilitators such as support networks have been identified [54, 70].

Potential strategies to improve adherence that have been considered include offering home‐based programmes or using digital technology to deliver interventions [71, 72]. However, home‐based or remotely monitored programmes must consider digital exclusion, risk assessment and mitigation for vulnerable groups (e.g. those at risk of falls), and the need for regular support, as home‐based interventions have low compliance and high attrition rates when patients feel unsupported [40, 69]. Other strategies to improve acceptability and adherence include goal setting; enhancing patients' confidence in their ability to engage; clarifying the purpose of the intervention; promoting social support; offering time‐efficient exercises; and enabling activity tracking [64, 66]. It has also been suggested that the provision of ‘patient‐centred’, multimodal prehabilitation interventions might improve adherence [69]. However, while multiple studies support the importance of personalised offers for patients, sometimes this represents personalisation of interventions to accommodate the physical condition of the patient, without also considering their values, wishes and cultural preferences, and therefore may fail to provide truly patient‐centred care [62, 64]. As many patients in qualitative studies report benefits of prehabilitation such as gaining a sense of control, support from others, and self‐perceived benefits to health, consulting with patients in the design and delivery of services and interventions is key [61].

Additionally, studies have highlighted health literacy as a barrier to adherence [3], particularly among individuals from socio‐economically deprived communities [58], in which some patients appear to have a limited awareness of the physiological and psychological toll of surgery [52].

### Communicating prehabilitation to patients

One aspect of prehabilitation that appears to be somewhat overlooked in the research literature is the communication of information to patients. Patients with cancer are frequently dissatisfied with information about treatment [73], and communication is complicated by time restraints, variation in patients' information needs and varying levels of health literacy, as well as a lack of tools to assess these [74]. Providing patients with ‘good’ information can promote a sense of control, manage expectations and enhance shared decision‐making, while also contributing to satisfaction with, and adherence to, treatment [73]. Those with fewer unmet information needs tend to have lower levels of anxiety and depression, and higher global and mental quality of life [75].

Access to prehabilitation interventions is lower for people from socio‐economically deprived communities and some minority ethnic groups, for whom health literacy may be a barrier [58]. Effective communication underpins effective cancer care [76] but there is little research on this element of prehabilitation. In a study on long‐term outcomes of prehabilitation for patients with oesophageal adenocarcinoma, it was reported that patients cited reluctance to participate due to worry that cardiopulmonary exercise testing (CPET; a component of the programme in question) would identify them as ‘unfit’ for surgery and that this would lead to withdrawal from curative pathways [77]. The authors expressed concern that some of the least fit patients may have declined prehabilitation due to their fear of the implications of CPET and that those who did not participate had significantly reduced survival. This suggests that some patients may miss out on what may be the greatest potential benefit of prehabilitation, i.e. to allow someone who is initially ‘unfit’ for surgery to be able to access its benefits. It has been noted that patients from low socio‐economic backgrounds had a poorer understanding of prehabilitation components and proposed benefits, which may contribute to lower rates of participation [58]. Communicating the goals of prehabilitation and its role in treatment pathways is clearly about much more than sharing information about the programme in question and may require the delivery of broader health education for some patients if communication is to be equitable.

Across research and grey literature, prehabilitation is positioned in myriad ways. Sometimes referred to as ‘prevention in action’ [78] and other times as an extension of the cancer care continuum, prehabilitation is also positioned occasionally by advocates as equally important as other treatments, such as chemotherapy and radiotherapy. However, these varied definitions of what ‘counts’ may contribute to poor conceptual clarity, which in turn may contribute to poor awareness and uptake of prehabilitation [6] or unclear communication with patients.

There is a tendency for public‐ and patient‐facing literature to promote the benefits of prehabilitation in ways that can obscure the contingencies on which positive outcomes rely or the uncertainty of evidence more broadly. In researching issues of inequality in prehabilitation, we have come across numerous examples where prehabilitation is presented unequivocally as an intervention which improves survival or reduces cancer recurrence [79, 80, 81]. Although these are perhaps examples of well‐intentioned motivational communication, seeking to enthuse and engage patients, and we do not discount the benefits prehabilitation may offer, such claims overstate the benefits shown by current evidence. Certainly, communicating the benefits of prehabilitation is seen as important to motivation [68], and key to capitalising on the ‘teachable moment’ thought to occur between diagnosis and surgery [10].

Premised as a window of opportunity in which patients are more receptive to making positive behavioural changes, the ‘teachable moment’ has been intuitively accepted by researchers and clinicians as an optimal time to motivate patients to improve their health [82]. However, it remains a somewhat unclear concept and usually focuses on tackling a single ‘unhealthy’ behaviour (such as smoking or alcohol cessation) rather than a multicomponent programme of complex behaviour change [83]. Moreover, capacity to engage with theoretical ‘teachable moments’ is mediated by the wider context of social inequality in which access to resources, information and support that might facilitate behaviour change is constrained by social position [40]. Claims that prehabilitation may lead to longer‐term behaviour change and improve population health are, as yet, unevidenced with not enough research on the long‐term outcomes of prehabilitation interventions [40].

### Towards more equitable prehabilitation before cancer surgery

Implementation of prehabilitation is fraught with inequalities and challenges that hinder its accessibility and effectiveness across diverse population groups. Socio‐economic disparities, regional funding differences and varying levels of clinical support create significant barriers to equitable prehabilitation services. Given that prehabilitation is a demanding intervention in terms of system resources and patients' time, resources and energy, and is implemented during a challenging period in patients' lives, care should be taken to ensure that information that is used to enrol patients is evidence‐based and reflective of the intervention they are likely to receive. This is likely to be challenging, considering that it also needs to be clear and easy to understand. Interventions that premise behaviour change via the ‘teachable moment’ should provide tailored and inclusive support and resources to the most disadvantaged patients to mediate intervention generated inequality. There is a risk that those most likely to engage with prehabilitation represent the most advantaged. As such, unless it is carefully implemented, prehabilitation carries the risk of widening health inequalities.

With these challenges in mind, we are currently conducting a research project called *Prehabilitation for Cancer Surgery: Quality and Inequality* (*PARITY* [84]) which seeks to work with patients, carers and healthcare professionals to find ways to describe, measure and assess the quality of prehabilitation services and identify best practice examples of how they are developed, funded and delivered, including how they address health inequalities. At the time of writing, we have recently embarked on a series of case studies to investigate how prehabilitation before cancer surgery is done in practice, and we look forward to sharing our findings in due course.
